# Breeding system and geospatial variation shape the population genetics of *Triodanis perfoliata*

**DOI:** 10.1002/ece3.9382

**Published:** 2022-10-08

**Authors:** Morgan Tackett, Colette Berg, Taylor Simmonds, Olivia Lopez, Jason Brown, Robert Ruggiero, Jennifer Weber

**Affiliations:** ^1^ Neuroscience Graduate Program University of Oklahoma Health Sciences Center Oklahoma City Oklahoma USA; ^2^ Division of Biological Sciences University of Montana Missoula Montana USA; ^3^ School of Biological Sciences Southern Illinois University, Carbondale Carbondale Illinois USA; ^4^ Department of Biology Southeast Missouri State University Cape Girardeau Missouri USA

**Keywords:** breeding system, cleistogamy, landscape genetics, phylogeography, population genetics

## Abstract

Both intrinsic and extrinsic forces work together to shape connectivity and genetic variation in populations across the landscape. Here we explored how geography, breeding system traits, and environmental factors influence the population genetic patterns of *Triodanis perfoliata*, a widespread mix‐mating annual plant in the contiguous US. By integrating population genomic data with spatial analyses and modeling the relationship between a breeding system and genetic diversity, we illustrate the complex ways in which these forces shape genetic variation. Specifically, we used 4705 single nucleotide polymorphisms to assess genetic diversity, structure, and evolutionary history among 18 populations. Populations with more obligately selfing flowers harbored less genetic diversity (*π*: *R* ^2^ = .63, *p* = .01, *n* = 9 populations), and we found significant population structuring (*F*_ST_ = 0.48). Both geographic isolation and environmental factors played significant roles in predicting the observed genetic diversity: we found that corridors of suitable environments appear to facilitate gene flow between populations, and that environmental resistance is correlated with increased genetic distance between populations. Last, we integrated our genetic results with species distribution modeling to assess likely patterns of connectivity among our study populations. Our landscape and evolutionary genetic results suggest that *T. perfoliata* experienced a complex demographic and evolutionary history, particularly in the center of its distribution. As such, there is no singular mechanism driving this species' evolution. Together, our analyses support the hypothesis that the breeding system, geography, and environmental variables shape the patterns of diversity and connectivity of *T. perfoliata* in the US.

## Graphical abstract

Both intrinsic and extrinsic forces work together to shape connectivity and genetic variation in populations across landscapes. We explicitly examined how these factors influence the landscape genetics of a widespread cleistogamous species. Our analyses support the hypothesis that breeding system, geography, and environmental variables all shape the patterns of diversity and connectivity of *T. perfoliata.*

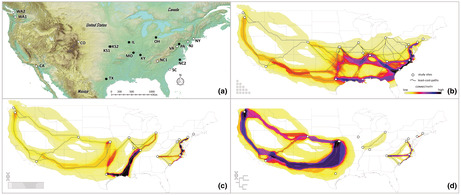

## 1 INTRODUCTION

Understanding what factors drive patterns of genetic diversity among populations is central to evolutionary ecology, and critical for predicting how species respond to changing environments (Manel & Holderegger, 2013). Evolution is intrinsically linked to genetic diversity, which often serves as the raw material for evolutionary processes (e.g., Alsos et al., 2012; Jump et al., 2009; Stange et al., 2021). However, data on quantitative traits are rarely available for wild species across their distributions, limiting our ability to study how traits evolve. Measures of genetic diversity from neutral markers are more readily available and correlations have been found between the differentiation of quantitative traits and neutral markers (e.g., Frankham et al., 1999; Jump et al., 2009; Merilä & Crnokrak, 2001). The adaptive potential may be limited in naturally occurring populations with low genetic diversity and small effective population sizes (Hobbs & Humphries, 1995; Jump et al., 2009; Lai et al., 2019; Lande & Shannon, 1996).

Intraspecific genetic diversity is often influenced by intrinsic factors such as variation in the reproductive system and demographic history (e.g., Chan et al., 2011; Clobert et al., 2012; Hellwig et al., 2021; Toczydlowski & Waller, 2019) and extrinsic factors such as interactions with barriers that limit dispersal (physical, abiotic, and biotic; e.g., Alvarado‐Serrano et al., 2019; Brown et al., 2016; Galbreath et al., 2010). Taken together, these factors shape the variation in gene flow among populations, influencing subsequent evolutionary processes (e.g., lineage diversification, hybridization) and patterns of genetic diversity among populations (e.g., Chan et al., 2011; Cruzan & Hendrickson, 2020; Hellwig et al., 2021). Studies that consider both intrinsic and extrinsic factors over multiple scales provide a more complete interpretation of what drives patterns of genetic diversity at the intraspecific level (Schregel et al., 2018; Twyford et al., 2020).

Because flowering plants often exhibit high intraspecific variation in reproductive systems, they present novel opportunities to examine the role of breeding systems in influencing patterns of genetic diversity and divergence among populations (e.g., Culley & Stokes, 2012; Sun et al., 2002; Toczydlowski & Waller, 2019). For example, many flowering plants have the capacity for both cross‐ and self‐fertilization, a condition termed mixed‐mating (Goodwillie et al., 2005; Lande & Schemske, 1985). Self‐fertilization presents several benefits in the context of mate availability and range expansion (Baker, 1955; Busch & Delph, 2012). Individuals within populations with relatively high inbreeding exhibit high genetic similarity, reducing effective population size. In turn, these populations are highly susceptible to genetic drift and subsequent loss of genetic diversity and potentially greater susceptibility to changes in the external environment (Lande, 1988; Lande & Shannon, 1996; Wright, 1946). Inbreeding, particularly in smaller populations, also drives differentiation among populations, in parallel with patterns expected for geographically isolated populations (Lowe et al., 2005; Toczydlowski & Waller, 2019; Wright, 1965). Despite the potential importance of plant reproduction in driving patterns of genetic diversity and connectivity, this feature of species is still poorly understood in the context of population genetics at large spatial scales in wild populations.

Among populations, a common explanation for spatial patterns of genetic diversity is isolation‐by‐distance (IBD), where populations that are geographically isolated exhibit greater genetic differentiation via attenuated gene flow and genetic drift (Wright, 1943, Slatkin, 1993, e.g., Toczydlowski & Waller, 2019, Hellwig et al., 2021). While greater inbreeding may drive genetic isolation at a local scale, IBD often has a greater influence at broad spatial scales. Larger populations are typically less susceptible to drift, but founder effects and population bottlenecks can still drive genetic differentiation in isolated populations, especially following colonization events (Toczydlowski & Waller, 2019; Wright, 1977). In addition to IBD, many studies invoke key roles for variation in topography and climate in mediating spatial distribution patterns, which either restrict dispersal (e.g., mountains, rivers, etc.), or act as suitable corridors for gene flow (isolation by the environment; McRae, 2006). Species also exhibit specific ecological tolerances that dictate spatial patterns of gene flow and migration (Chan et al., 2011; Sexton et al., 2014; Wang & Summers, 2010). Across heterogeneous landscapes, areas of ecological tolerance for a species may be more limited, resulting in increased genetic divergence among populations by reducing dispersal corridors (Wang & Bradburd, 2014). Incorporating estimates of environmental tolerance with IBD provides a more realistic framework for understanding population connectivity across landscapes (e.g., Alvarado‐Serrano & Hickerson, 2018; Cruzan & Hendrickson, 2020; Cushman et al., 2009; Hevroy et al., 2018; Toczydlowski & Waller, 2019; Wang & Bradburd, 2014).

Integrating genetic data with landscape and environmental parameters can better describe the range of factors driving or maintaining patterns of genetic diversity among populations (e.g.Alvarado‐Serrano & Hickerson, 2018; Chan et al., 2011; Cruzan & Hendrickson, 2020). Here we explicitly examine how breeding system variation, geographic distance, and habitat suitability may be integrated to explain spatial patterns of genetic diversity in 18 populations of *Triodanis perfoliata* (Campanulaceae), a widespread, annual native to North and South America (Weakley, 2010). All individuals of this species exhibit dimorphic cleistogamy, consisting of both obligately self‐fertilizing flowers and flowers that can either self‐ or cross‐fertilize (Gara & Muenchow, 1990; Trent, 1942). Because of the high potential for inbreeding in populations of *T. perfoliata*, we also examine how breeding system may correlate to metrics of genetic diversity and influence overall patterns of genetic structure. At broad geographic scales we predict high levels of population structure and relatively high population genetic divergence; at this scale we predict that both isolation by distance (IBD) and isolation by environment (IBE) will be the strongest factors structuring genetic diversity. Both geographic distance and variance in biotic and abiotic factors can limit gene flow or shape the potential for gene flow through particular corridors. Therefore, we explicitly discern the roles of geographic isolation (IBD) and environment (IBE) in shaping observed patterns. At local spatial scales, we predict that populations with a greater allocation to cleistogamy will exhibit reduced genetic diversity and high population genetic structuring (average *F*_st_) due to increased inbreeding and that the breeding system will be a more important factor influencing population‐level genetic patterns. Finally, we use habitat suitability models to predict routes of dispersal among contemporary populations. Incorporating genetic data into these analyses provides a framework for understanding corridors of gene flow among our study populations.

In concert with our other predictions, we expect the models to reflect limited gene flow among geographically or environmentally isolated population genetic clusters and phylogenetic clades; and that some genetic groups may appear genetically isolated despite the potential for gene flow through these corridors, due to increased selfing or other ecological factors. We analyze both population‐ and evolutionary genetic relationships to better understand the contemporary and historical connectivity among populations. Overall, we aim to outline a thorough framework of factors driving observed population genetic patterns at both broad and narrow scales.

## 2 METHODS

### 2.1 Study species & breeding system

*Triodanis perfoliata* (L.) Nieuwl. (Campanulaceae) is a small, common, annual herb native to North and South America. This weedy annual grows in a variety of conditions including disturbed areas, along rocky outcrops, dry open habitats, and prairies (Gleason & Cronquist, 1991; Weakley, 2010). Seeds of this species are quite small (approx. Length = 0.5 mm, width = 1.3 mm) and may be dispersed by ants (McVaugh, 1948; Shetler & Morin, 1986). Individuals exhibit a mixed mating system via dimorphic cleistogamy that includes two distinct floral types. Chasmogamous (CH) flowers are purple, five‐petaled, ~1.5 cm in diameter, and can either outcross or self‐fertilize; cleistogamous (CL) flowers completely lack a corolla and are obligately self‐fertilizing (Gara & Muenchow, 1990; Goodwillie & Stewart, 2013; Trent, 1940). All individuals of *T. perfoliata* exhibit both floral types and there is considerable variation among populations in the relative production of CH to CL flowers (Ansaldi, Franks, & Weber, 2018). Some of this breeding system variation is driven by variation in pollination visitation and abiotic conditions (Ansaldi, Franks, & Weber, 2018; Ansaldi, Weber, & Franks, 2018).

### 2.2 **DNA** collection, extraction, and sequencing

**FIGURE 1:**
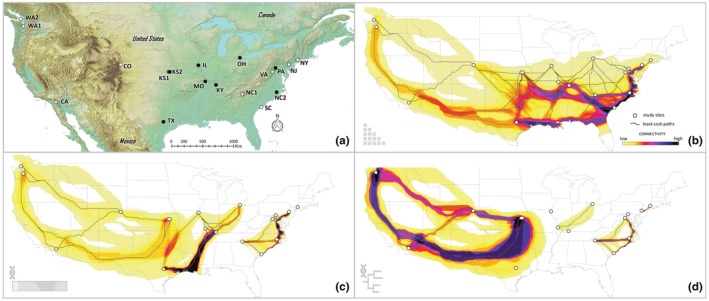
Sample localities and models of population connectivity. (a) Study site localities (*n* = 18); filled markers indicate sites for which breeding system traits were estimated (*n* = 9). (b) Population connectivity among all sites (c) population connectivity among genetic clusters (*k* = 4). (d) Population connectivity among major phylogenetic clades. Dark lines depict least‐cost paths. Groups with no connections represent either clades or cluster groups that exist only at that locality.

In late spring and early summer 2017, leaf tissues were collected in the field from 18 populations of *T. perfoliata* (total = 76 individuals; range = 1–6 individuals/population) spanning the contiguous US (Figure 1a), and from 6 individuals of *T. biflora* from southeast Missouri (Midwestern US) to serve as an outgroup for phylogenetic analyses. We used a CTAB protocol (Doyle & Doyle, 1987) to extract high quality genomic DNA from silica dried leaf tissue. Subsequently, RADSeq (Restriction site Associated DNA Sequencing) was performed at Floragenex, Inc. to identify genetic variants (Eaton, 2014). The restriction enzyme Sbf1 generated short fragments prior to the addition of sequencing adapters, and all samples were analyzed on the same flow cell with Illumina 1x91bp sequencing. After sequencing, quality control and sequence alignment were conducted using Bowtie (Langmead & Salzberg, 2012), BWA (Li, 2011) and Velvet (Zerbino, 2010) and variant calling were performed using Samtools (Li et al., 2009). The final dataset consists of variant calls with a minimum sequencing depth of 15x, minimum Phred score of 20, and no more than 10% of missing genotypes.

A total of 9,716,774 raw reads were generated, of which 9,657,413 passed quality filters. These were used to build 5,646,126 provisional clusters, i.e., groups of sequencing read that likely cover the same position in multiple samples, each with a minimum cluster depth of 5x and maximum cluster depth of 1500x. After reading alignment and quality assessment, this yielded a final assembly that was approximately 5.2 Mb in length, consisting of 56,6649 contigs, each with a length of 92 bp. An average of 38.9% of the sequence reads from each sample aligned to a single position in this assembly. Variant calling yielded 4705 single nucleotide polymorphic (SNP) sites observed >90% of the sequenced individuals of *T. perfoliata*.

### 2.3 Genetic diversity & population structure

Bayesian cluster analyses were performed using STRUCTURE v2.3.4 (Pritchard et al., 2000). Ten independent runs were performed for each potential number of genetic clusters (K) [value 3–22] using a burn‐in period of 40,000 and followed by 80,000 iterations per *K*; analyses were run under the admixture model and assuming correlated allele frequencies. To determine the most likely value for *K*, we assessed values of Δ*K* (evaluating the second‐order rate of change of the likelihood function), as per the Evanno et al., 2005 method in Structure Harvester v.6.0 (Earl & vonHoldt, 2012). Global *F*_ST_ was calculated via the R packages Adegenet (Jombart & Ahmed, 2011) and Hierfstat (Goudet, 2005); the R package vcfR (Knaus & Grünwald, 2017) was used for file conversion. Genetic divergence between populations and genetic clusters (pairwise *F*_ST_, Tajima & Nei, 1984) and population level statistics (i.e., number of private alleles, *π* [mean number of pairwise differences per site], number of polymorphic sites) were calculated using Arlequin 3.5.2.2 (Excoffier & Lischer, 2010).

### 2.4 Phylogenetic tree estimation

We used RAxML V8 (Stamatakis, 2014) to create a maximum likelihood phylogeny from over 6000 SNPs. Phylogenetic trees were generated using *ASC_GTRGAMMA* model of nucleotide evolution, which is an ascertainment bias general‐time‐reversible model (Lewis, 2001). Phylogeny support was estimated by using 10,000 rapid bootstrapped trees. Direct confirmation was conducted by splitting the data set into five subsets, each consisting of 1200 SNPs, and generating phylogenies using the same parameters as the complete data set to ensure the absence of major deviations in the resulting inferences.

### 2.5 Reproductive system assessment

Following methods in Ansaldi, Franks, & Weber, 2018, we quantified the breeding system (i.e., extent of cleistogamy) in a subset of populations included in our genetic analyses (Figure 1a: filled circles). Because these analyses aimed to estimate the total floral input of each flower type in a population (total CH and CL), we used only individuals with fully mature stems (flowering completed), and populations for which we had access to *N* ≥ 20 vouchered individuals. With these limitations, we assessed the breeding system for *N* = 9 of our 18 overall populations with samples from 2017 (the same year as tissue collections for population genetic analyses). Breeding system data for the OCN population (Otter Creek North Carolina) were derived from Ansaldi, Franks, & Weber, 2018. The total average production of each flower type in each population was estimated by collecting whole individual, fully mature plants (range = 20–50; 33 = mean individuals per population). For each population, we assessed the average number of CH flowers, number of CL flowers, total flower number and the proportion of flowers that were CH out of the total flower number (pCH). To test the hypothesis that populations with a greater allocation to CL flowers will exhibit greater overall population structuring, we performed a linear model between pCH and mean population pairwise FST and to test the hypothesis that populations exhibiting greater proportional production of CL flowers may maintain less genetic diversity, we performed linear regressions between metrics of genetic diversity (e.g., *π*, number of polymorphic sites) and pCH via a linear regression using the *lm* function in R statistics v 4.1.1 (R Core Team, 2021).

### 2.6 Predicting dispersal networks

#### 2.6.1 Creating the SDM

Likely routes of dispersal among populations or genetic groups were predicted via least‐cost corridor analyses, an approach that incorporates species distribution models (SDMs; Chan et al., 2011). SDMs were generated using occurrence records collected between the years 2000–2019 obtained from digital herbarium vouchers, primary literature, our lab fieldwork, and open‐source occurrence data (*n* = 4503 initial records; GBIF.org 2020; *addn*. Data and citations available in Berg et al., 2019). Data were first vetted for taxonomic assignment as well as apparent labeling errors (e.g., data points in oceans). Spatial clusters of localities can cause models to over‐fit toward environmental biases and inflate model performance values (Boria et al., 2014; Hijmans, 2012; Veloz, 2009). Spatial biases were addressed by randomly selecting points clustered within a 10‐km radius using SDMtoolbox 2.4 (Brown, 2014). The final vetted dataset consists of 1735 occurrence records. Nineteen bioclimatic layers at a 30 arc‐minute resolution from WorldClim v2.0 (Hijmans et al., 2005) were used to generate species distribution models (SDM) in MaxEnt 3.3.3k (Phillips et al., 2020). SDMs were parameterized with SDMtoolbox v2.4 (Brown, 2014), to evaluate the performance of various combinations of five feature classes (linear; linear and quadratic; hinge; linear, quadratic and hinge; and linear, quadratic, hinge, product and threshold), and five regularization multipliers (0.5, 1, 2, 3, 4; Radosavljevic & Anderson, 2014). SDM performance built under each combination of parameters was assessed through a geographically structured *k*‐fold cross‐validation (i.e., the occurrence records were partitioned into *k* equal geographically clustered subsamples, here *k* = 3, and the models were trained with two of the groups and then evaluated with the excluded group until all group combinations were run). Model fit was assessed via the omission rate, area under the curve (AUC), and model feature class complexity (Brown, 2014). After optimum model parameters were determined (those leading to the lowest omission rate, highest AUC, and lowest complexity, in the order listed), a final SDM was built with all occurrence sites and projected into the current climate across the contiguous US, southern Canada, as well as northern Mexico.

The final SDM estimates contemporary habitat suitability and was used to estimate potential dispersal networks among populations and genetic groups of *T. perfoliata* in our genetic analyses. These Least‐Cost Corridors (LCCs) are estimated by inverting the SDM (one minus SDM suitability values) to function as a friction layer, characterizing the cost of dispersal through each pixel in the landscape; areas of high suitability have a lower dispersal cost compared to areas of low suitability (Chan et al., 2011). We examined multiple separate scenarios to understand how connectivity among these populations may influence patterns of genetic diversity.

#### 2.6.2 Population connectivity (SDM only)

In the first scenario, dispersal networks were estimated between all 18 populations included in our genetic analyses. This model serves as a null hypothesis by solely considering how habitat suitability predicts population connectivity in the absence of genetic data. Connectivity among genetic clusters. In the second scenario, dispersal networks were estimated among the genetic groups described in analyses of genetic structure (most likely *K* value). This scenario describes the role of likely dispersal corridors in shaping the genetic structure seen across the sampled landscape. Connectivity among clades. In the third scenario, dispersal networks were estimated among major phylogenetic clades. Here we subjectively split the phylogeny into subclades by placing a vertical line near the base of the tree (see dashed line in Figure 3), which split the phylogeny into 8 evolutionary groups that each share common ancestry with clade members. These sub‐clade groupings were chosen, in part, because they matched our structure groups (though sub‐divided due to discordance in our results) and each sub‐clade was assigned entirely to the same cluster group (with the exception of the clade containing the NY cluster). Decreasing the sub‐clade group number (by moving the vertical line toward to most‐recent common ancestors) would have resulted in more clades containing mixed cluster groups, whereas increasing group‐number would have removed deeper evolutionary relationships into dispersal corridors calculation.

### 2.7 Examination of IBD and IBE

To quantitatively test the relationships between the observed genetic divergence and both IBD and IBE, we used Multiple Matrix Regression with Randomization (MMRR) analyses in R (see Wang et al., 2013 for scripts to perform analysis). For these analyses, we first generated the following four distance matrices: genetic distance, geographic distance, environmental least‐cost path distance, and environmental least‐cost path total resistance. Genetic distance was quantified by measuring the inter‐population FST among the 18 research sites in Arlequin v3.5 (Excoffier & Lischer, 2010). Geographic distance was calculated by measuring the Euclidian distance between the research sites. To investigate the explicit role of IBE, we calculated the least‐cost paths among the 18 research sites using our final SDM, a friction layer where the suitability values were inverted (Chan et al., 2011). This analysis resulted in the creation of two distance matrices: (1) a matrix measuring the path length of the least‐cost paths and (2) a matrix measuring the total resistance cost of the least‐cost paths among the research sites. All spatial measurements and analyses were performed in ArcGIS 10.7 (ESRI, 2021) using SDMtoolbox v2.4 (Brown et al., 2017). The two raw IBE matrices, distance and resistance values, were highly correlated with the IBD matrix (*R* ^2^ = .988 and *R* ^2^ = .914, respectively). To remove the explicit effects of geographic distance from our two IBE matrices, we performed a linear regression in which each IBE distance matrix was a response variable and our geographic distance matrix was the predictor variable (Davies et al., 2007; Fritz & Rahbek, 2012; Vale et al., 2018). We used the resulting residuals output from each linear regression, one where environmental least‐cost path (LCP) distance was input and a second where the environmental LCP total resistance was input (correlation to IBD matrix: *R* ^2^ = .156 and *R* ^2^ = .406, respectively), as our corresponding IBE distance matrices in separate MMRR analyses. In each MMRR analysis, genetic distance was used as a response variable, whereas the IBD and IBE matrices were each considered individually as a single predictor variable. To assess the statistical significance of each MMRR analysis and predictor variables, we used 10,000 permutations in each analysis (Wang et al., 2013).

## 3 RESULTS

### 3.1 Genetic diversity and population structure

RADSeq yielded approximately 3.8 million bases and *n* = 631,465 total genotypes. We found *n* = 4705 single nucleotide polymorphisms (SNPs) in aligned sequence sites for *T. perfoliata*. These SNPs were presumably random except for their proximity to the Sbf1 restriction site that was used to generate the library. There is no reason to believe there is systematic selection occurring on the eight bases that constitute the Sbf1 target sequence (CCTGCAGG). The final, stringent list of variants contained data for 97% of genotypes; 3% of genotypes were not sequenced or found to have low quality, and therefore were considered missing. Total heterozygosity was relatively low (16%) and a potential signature of high inbreeding in this species. The number of private alleles, *π*, and polymorphic sites varied widely among the 18 populations, indicating considerable variation in genetic diversity (Table 1). Pairwise *F*_ST_ revealed a considerable range in genetic differentiation between populations and genetic clusters; population pairwise *F*_ST_ range (0.01–0.80) (Tables S1 and S2). In addition, global *F*_ST_ = 0.48 was quite high suggesting substantial overall population genetic differentiation which may be driven by the capacity for inbreeding in this species.

**TABLE 1:** Characteristics of 76 individuals from 18 populations from across the contiguous US

Pop	No private alleles	*π*	No poly sites	Individuals	pCH	Long (DD)	Lat (DD)
CA	35	403.30	379	415		−116.614	33.680
CO	90	509.62	728	323, 324		−105.112	40.352
IL	221	555.92	1149	158, 161, 163, 165, 170, 173	0.53	−91.242	40.218
KS1	66	552.69	1056	176, 179, 180, 183, 191	0.76	−96.593	39.095
KS2	225	660.53	1480	196, 200, 201, 205, 209, 240	0.74	−96.617	39.095
KY	304	655.96	1229	101, 105, 111, 112, 118	0.52	−88.117	36.734
MO	66	220.82	352	446, 448, 450	0.27	−90.023	37.358
NC1	79	381.45	999	81, 84, 88, 91, 92, 94		−83.431	35.060
NC2	393	709.87	1592	226, 227, 230, 233, 234, 239	0.58	−77.310	35.431
NJ	103	448.20	906	242, 243, 245, 246, 250		−75.112	40.361
NY	16	195.77	352	440–445		−73.574	41.208
OH	17	245.34	353	313, 318	0.40	−83.852	41.555
PA	16	196.79	347	219–222, 224	0.16	−77.501	39.732
SC	20	377.58	356	373		−80.040	32.788
TX	238	493.56	863	39, 40, 43, 44, 47	0.33	−97.466	30.170
VA	61	468.26	882	65, 67, 71, 75, 77, 78		−78.065	39.063
WA1	61	205.59	345	405–407, 409		−122.444	47.144
WA2	103	243.34	346	330, 336		−122.903	48.447

*Note*: By population: Number of private alleles, *π*, number of polymorphic sites generated in Arlequin. For nine populations, pCH indicates an estimate of the population breeding system (average proportion of CH flowers to CL flowers); *addn*. Breeding system information: Table S3. Longitude and latitude measured in decimal degrees.

**FIGURE 2:**
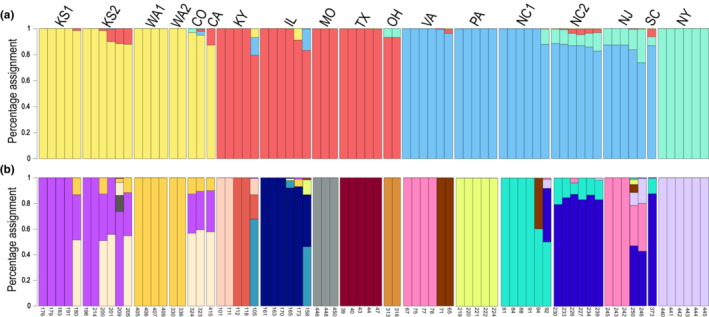
Results from population structure analyses showing (a) *K* = 4 and (b) *K* = 17; two scenarios of sub‐structuring with the highest likelihood values using the Evanno et al. (**2005**) method in STRUCTURE Harvester. Letters above the bars indicate the population (by US state, numbers for multiple populations in a single state); numbers below the bars indicate the individual identifier. Colors from the *K* = 4 plot are replicated in Figure 3 to show discordance between structure analyses and phylogenetic results.

Analyses of the genetic structure revealed high support for both *K* = 4 and *K* = 17 genetic clusters among the 18 populations of *T. perfoliata* (Figure 2). For *K* = 4 genetic clusters, which had the highest likelihood support values (e.g., Δ*K* = 7.68), populations generally segregated into broad geographic regions of the US including (1) the central Midwest and Western states, (2) the southern Midwest and Gulf states, (3) Eastern states and (4) populations from NY that form a separate cluster (Figure 2a; Figure S2b). At *K* = 17 genetic clusters (Δ*K* = 6.28), individuals generally segregated into clusters by population (Figure 2b; Figure S2c). But similar to the *K* = 4 profile, within the *K* = 17 structure profile, some populations from the central Midwest and Western US shared genetic clusters (i.e., CA, CO, KS1, KS2), and some populations from the Eastern US also clustered together (e.g., SC, NC2, NJ Figure 2b). We also accessed genetic clustering using the package sMNF (Frichot et al., 2014). sMNF is robust to departures from population genetic assumptions; results were overall very similar to our results from STRUCTURE (Figure S1).

### 3.2 Phylogenetic tree estimation

**FIGURE 3:**
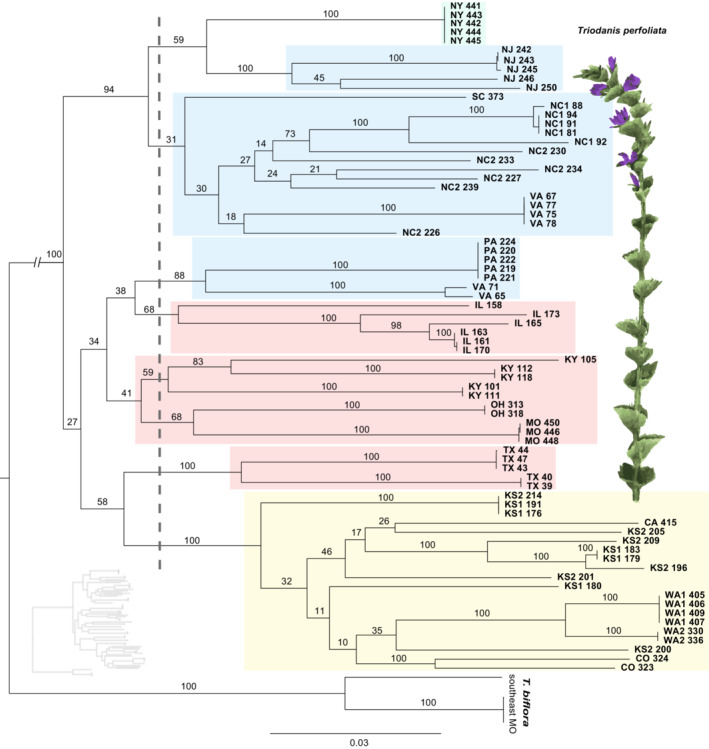
RaxML maximum likelihood phylogenetic tree. Colors boxes indicate primary individual identity to genetic clusters corresponding to the plot *K* = 4, (Figure 2). Letters indicate the population (by US state, numbers for multiple populations in a single state); numbers indicate the individual identifier. Truncated branch length at base has a length of 0.0603. Small inset tree is a tree without basal nodes trimmed. Branch lengths and scale bar refer to the number of nucleotide substitutions per site. Inset image is modified from a hand‐colored lithograph by Endicott based on an illustration from John Torrey's *a Flora of the state of New York* (Torrey, **1843**).

Phylogenetic analyses revealed three major geographic clades of *T. perfoliata* within the US (Figure 3). A couple of interesting phylogenetic patterns concur with specific results from genetic clusters from STRUCTURE: populations from KS and CO share a clade with populations from CA and WA, despite the significant geographic distance between these sites. In addition, individuals from a population in VA appear in both the clade consisting primarily of Eastern state populations and the clade of Midwestern and Gulf state populations. But overall, our phylogeny shows discordance with results from STRUCTURE (see colored boxes in Figure 3 correspond to colors associated with *K* = 4 cluster Figure 2a). Only two genetic clusters, NY and the KS, CO, CA, WA cluster form monophyletic groups. We acknowledge that some bootstrap support values are relatively low, particularly among individuals from the Midwest, whereas the coastal populations generally have higher support. In general, cluster groups with mixed group assignment (*K* = 17 Figure 2b) also had much lower support in our phylogenetic trees (e.g., compare the mixed cluster groups KS2 and NC2 with their corresponding phylogenetic support values).

### 3.3 Reproductive system assessment

**FIGURE 4:**
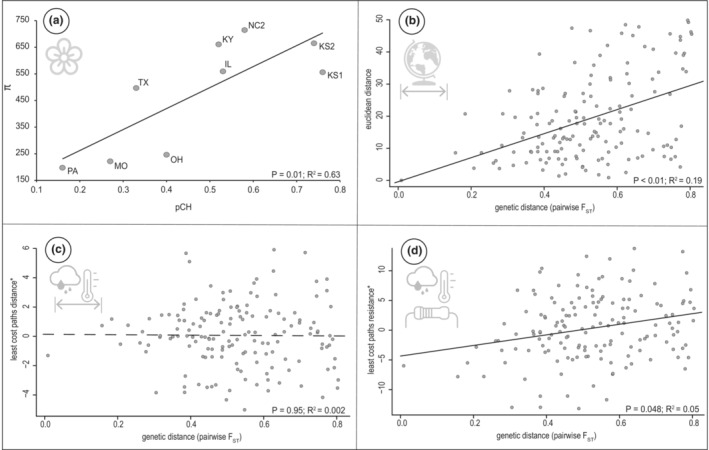
(a) Correlation between breeding system (estimated as the average proportion of cleistogamous to chasmogamous flowers produced among individuals in a population: pCH) and genetic diversity (*π*: The number of nucleotide differences per site between two randomly chosen sequences from a population) in *n* = 9 populations. MMRR correlations between genetic distance and: (b) Euclidian distance (in decimal degrees), (c) environmental least‐cost path distance, (d) environmental LCP total resistance. *For both (c) and (d), the matrices analyzed represented residuals of a lineage regression of the raw environment matrix and geographic distance. This removed the effect of geographic distance from these MMRR analyses.

Similar to previous work, we found that breeding systems are highly variable across populations of *T. perfoliata* (i.e., mean relative chasmogamy (pCH) was 0.48 ± 0.07 [*N* = 9; mean ± 1 SE; Table S3]). Across these nine populations, mean population pairwise *F*_ST_ was significantly negatively correlated with pCH; populations producing relatively more CL flowers exhibited greater overall population substructuring (*p* = .0015, multiple *R* ^2^ = .78, *F* = 25.19; Table S4). In addition, the relative proportion of CH flower production (pCH) is significantly positively correlated to multiple metrics of genetic diversity (*π*: *R* ^2^ = .63, *p* = .01, Figure 4a; number of polymorphic sites: *R* ^2^ = .63, *p* = .01). These strong associations support the hypothesis that breeding system strongly influences population genetic patterns. Populations with a greater allocation to CL flowers exhibit reduced genetic diversity and increased population substructuring, as predicted by reduced gene flow among highly selfing populations.

### 3.4 Predicting dispersal networks

Our final SDM had a high predictive performance with an Omission Rate (OR) < 0.01 and a moderate discriminatory ability with an AUC = 0.62 (Supp. Figure 2). The resulting distribution prediction matches our expert knowledge of the species' distribution. Population connectivity (SDM only). Our first LCC model considered only the role of habitat suitability in influencing dispersal corridors among our study populations (Figure 1b). Without the inclusion of genetic data, the LCC model assumes an equal probability of connectivity among all of our 18 populations. In this model, we observe many potential dispersal corridors among Midwestern, Gulf, and Eastern states within the US; and multiple potential dispersal corridors between the Western US and the Midwest and Gulf states, but the most highly predicted corridor goes through the Southwestern US (Figure 1b).

#### 3.4.1 Incorporating genetic information in LCCs

To better visualize the likely dispersal corridors among groups, we incorporated genetic data into the LCC corridor calculations using two methods. Connectivity within population genetic clusters. The first method, the analysis of potential corridors among *K* = 4 genetic clusters, differs greatly from the null model (which compared all populations in LCC creation). Using genetic clusters imposes key roles for factors such as demography and lineage history in shaping the genetic landscape in addition to IBD and IBE (Figure 1c). In this model, the NY population becomes isolated, and no dispersal corridors are predicted between the Eastern states and Midwest and the Western US states. In this same model, multiple dispersal corridors between the Midwest and Western US are predicted as suitable, but the Southwestern corridor route still appears most likely. Interestingly, this model strongly predicts suitable dispersal between the Gulf states and several Midwest states (e.g., IL, OH, KY, MO). (Analyses of dispersal corridors among K = 17 genetic clusters were not logical because individuals from populations frequently belonged to multiple genetic clusters, resulting in relatively few localities to model.) Connectivity within clades. Our second genetic‐based LCC analysis incorporated phylogenetic relationships in the LCCs calculation. To do this we estimated likely dispersal corridors among major phylogenetic clades from our RaxML analyses (see vertical dashed line Figure 1d). This LCC model highly predicts dispersal between the Midwest and Western US States.

### 3.5 Examination of IBD and IBE

Geography (or IBD) was the strongest predictor of our observed genetic diversity across the sampled distribution (*R* ^2^ = .189, *p* < .01; Figure 4b). However, environment with the signal of IBD removed, also significantly predicted the observed genetic diversity (IBE distance, *R* ^2^ < .001, *p* = .945, Figure 4c; IBE total resistance *R* ^2^ = .058, *p* = .048, Figure 4d), however with a lower *R* ^2^ when compared to IBD.

## 4 DISCUSSION

Here we explicitly demonstrate how a variety of factors, both intrinsic (i.e., breeding system variation) and extrinsic (i.e., IBD, IBE), drive patterns of genetic diversity and population divergence in the mixed‐mating annual, *Triodanis perfoliata* (Campanulaceae). Specifically, we found that populations with a greater proportion of cleistogamous (obligately selfing) flowers had reduced genetic variation compared to populations with relatively more open flowers (*π*: *R* ^2^ = .63, *p* = .01). Greater inbreeding also tends to increase population structure and reduce gene flow among populations (Lande, 1988; Lande & Shannon, 1996; Wright, 1946). In concert with these predictions, we also found a strong negative association between mean population pairwise *F*_ST_ and the extent of open flowers (CH) produced in populations; suggesting greater gene flow among populations with a greater allocation to CH compared to CL (closed) flowers. Overall this species exhibits a relatively high global *F*_ST_ (0.48), a wide range of population pairwise *F*_ST_ values among populations and genetic clusters (Tables S1 and S2), and significant genetic sub‐structuring (highest likelihood *K* = 4 out of 18 populations). Genetic divergence among populations of *T. perfoliata* is also influenced by both geographic distance and environmental factors. Therefore, we also explored models of population connectivity to better understand the factors shaping the genetic landscape of this widespread, mixed‐mating species.

Reproductive systems can influence population dynamics in complex ways, driving patterns of genetic diversity, gene flow, and demographics, and influencing fitness (e.g., Charlesworth & Charlesworth, 1987; Lande & Shannon, 1996; Wright, 1946). Our findings are consistent with some studies that have also found significant population genetic structuring in dimorphic cleistogamous species (e.g., Lesica et al., 1988; Schoen, 1984; Sun et al., 2002; Toczydlowski & Waller, 2019). However, some cleistogamous species can also exhibit relatively high levels of genetic diversity and low population genetic structure (e.g., Cortés‐Palomec et al., 2006; Culley & Wolfe, 2001). These patterns demonstrate that the impact of cleistogamy on population genetic patterns is context‐dependent because some populations can still exhibit high levels of outcrossing in chasmogamous flowers (Culley & Wolfe, 2001).

Another important feature of cleistogamous species, including *T. perfoliata*, is plasticity in reproductive allocation to cleistogamy due to abiotic and biotic factors (Ansaldi, Franks, & Weber, 2018; Ansaldi, Weber, & Franks, 2018; Jones et al., 2013). In this study, we took advantage of population‐level variation in the breeding system to address if greater average cleistogamy impacts population genetic diversity in *T. perfoliata*. We acknowledge that in this study we did not directly link the breeding system to quantitative estimates of inbreeding (F_IS_). However, strong correlations between the extent of cleistogamy at the population level and metrics of genetic diversity suggest a meaningful relationship that warrants subsequent investigation. Inbreeding depression is an obvious potential negative consequence of greater inbreeding (Charlesworth & Charlesworth, 1987; Culley & Klooster, 2007), but our previous work demonstrated relatively low inbreeding depression for three populations of *T. perfoliata* under greenhouse conditions (Ansaldi et al., 2019). Nonetheless, here we demonstrate that populations with more average cleistogamy harbor less genetic diversity and reduced genetic diversity may result in long‐term demographic consequences that are more difficult to quantify on short time scales (Hobbs & Humphries, 1995; Jump et al., 2009; Lai et al., 2019; Lande & Shannon, 1996). Variation in relative production of cleistogamy in *T. perfoliata* is influenced by a variety of factors including soil type, water availability and pollinator environment (Ansaldi, Franks, & Weber, 2018; Ansaldi, Weber, & Franks, 2018). Taken together, dimorphic cleistogamy presents a novel opportunity to understand the multifaceted nature in which reproductive systems and ecology influence population genetic patterns.

Increased inbreeding, geographic distance, and environmental resistance will all lead to population divergence and strong patterns of population sub‐structuring. For *T. perfoliata*, we found evidence of high population sub‐structuring and strong geographic signals describing regional connectivity among our study populations. Mixed‐matrix models revealed that both geographic distance and environmental resistance describe significant patterns of population genetic divergence. Sexton et al. (2014) surveyed a wide range of taxa and found that IBE was the strongest pattern observed among animals, but both IBD and IBE were the strongest patterns among plants. Geographic distance is likely to incorporate multiple environmental factors, whereas IBE is typically limited to the scope of factors included in analyses. We explicitly accounted for IBD in our analyses of IBE and found that isolation by environment and genetic distance were correlated, suggesting that environmental resistance can lead to increased genetic divergence between populations, likely through a combination of drift and local adaptation. The variety of mechanisms potentially driving IBE are quite diverse (e.g., natural selection, phenology), but our data suggest that suitable corridors of population connectivity play a key role in facilitating gene flow between populations of *T. perfoliata*.

We modeled connectivity among all populations, as well as genetic groups to elucidate how various factors interplay to shape broader landscape genetic patterns for *T. perfoliata*. For these models, routes of dispersal are predicted using least‐cost corridor (LCC) analyses, a method that uses SDMs to find predicted corridors (Chan et al., 2011). As a null model, we first predicted dispersal corridors among all of our 18 study populations in the absence of additional genetic information (Figure 1b). This type of model upweights the relationships among distantly related individuals, which typically are more geographically isolated. Because of the indiscriminate nature of these types of analyses, a landscape can be hyper‐connected, with all populations being connected via corridor pathways. Such a landscape ignores spatial‐temporal dynamics associated with historical habitat and climate change (i.e., glacial cycles or climatic refugia). To build a more realistic model of landscape connectivity, we next incorporated genetic relationships into our landscape connectivity model. We modeled population connectivity among *K* = 4 genetic clusters, and this model was markedly different from the null model (Figure 1c). In the genetic cluster model, no dispersal corridors are predicted to connect with the NY population, even with geographically close populations. In other areas, we see broad patterns, predicting high connectivity in areas of high habitat suitability (Midwestern US) and reduced connectivity for geographically distanced localities, likely due to isolation by distance. This model also seems to account for barriers to dispersal, particularly the Appalachian and the Rocky Mountains, which either limit or reduce connectivity to the Midwestern US. We further explored how demographic and lineage history can influence genetic connectivity by integrating phylogeny into our analyses (Figures 1d and 3).

Due to methodological differences between our phylogenetic analyses and genetic clusters, the phylogenetic results aim to elucidate evolutionary relationships among populations and should clarify geographic regions that were historically connected at deeper timescales. In most cases, discordance between our structure and phylogenetic results (Figures 2 and 3) seem to reflect cases in which recent and frequent gene flow is likely between geographically close sites (i.e., admixture). Some of this discordance between our population genetic analyses and our phylogenetic analysis might be indicative of more complicated evolutionary histories, with isolated evolutionary lineages coming back into contact periodically. The low observed phylogenetic support values in the Midwest could be a result of such phenomena, though such hypotheses require proper statistical and population genomic analyses (see Figure 3; e.g., TreeMix, Pickrell & Pritchard, 2012). This is further reinforced by the differences between our phylogenetic LCC (Figure 1d) and our genetic cluster LCC (Figure 1c). Most notable is a lack of connection between Midwestern US populations on either side of the Mississippi River in the phylogenetic analyses, whereas populations are connected in the cluster LCC.

Our study encompasses a wide geographic area, and our data reflects both the implications of factors more predominant at the population level (e.g., breeding system) and factors affecting landscape‐scale patterns such as geographic distances (Husband & Barrett, 1996; Slatkin, 1987). For these reasons, future work will explore how current landscape genetic patterns may have been influenced by historic climatic patterns that may limit or facilitate population connectivity. For example, research will incorporate paleoclimate data with time‐calibrated phylogenies to estimate temporally relevant connections (for example see Guillory & Brown, 2021; French & Brown, 2022 *in preparation*). In addition, ongoing work aims to quantify how the breeding system explicitly influences patterns of demography and fitness among populations. Overall, our analyses of *T. perfoliata* illustrate the influence of the breeding system, geography, and the environment in shaping population genetic patterns in this widespread, mixed mating wildflower.

## AUTHOR CONTRIBUTIONS

**Morgan Tackett:** Conceptualization (equal); data curation (equal); formal analysis (equal); investigation (equal); methodology (equal); project administration (equal); writing – original draft (lead); writing – review and editing (equal). **Colette Berg:** Conceptualization (equal); data curation (equal); investigation (equal); project administration (equal); writing – review and editing (equal). **Taylor Simmonds:** Writing – review and editing (supporting). **Olivia Lopez:** Data curation (supporting); investigation (supporting). **Jason Brown:** Conceptualization (supporting); formal analysis (equal); methodology (equal); writing – original draft (supporting); writing – review and editing (equal). **Robert Ruggiero:** Data curation (equal); formal analysis (supporting); writing – original draft (supporting). **Jennifer Weber:** Conceptualization (equal); data curation (equal); formal analysis (equal); funding acquisition (equal); investigation (lead); methodology (equal); project administration (lead); supervision (lead); visualization (equal); writing – original draft (equal); writing – review and editing (equal).

## CONFLICT OF INTEREST

The authors claim no conflict of interest

## FUNDING INFORMATION

This work was supported by startup funds from Southern Illinois University, Carbondale to J. Weber, and additional research and startup funds from Southeast Missouri State University to J. Weber and R. Ruggiero.

## Supporting information


Table S1

Table S2

Table S3

Table S4

Figure S1

Figure S2

## Data Availability

Genomic data and breeding system data generated for this project are available via Dryad (https://doi.org/10.5061/dryad.sf7m0cg98 and https://doi.org/10.5061/dryad.wh70rxwr9).
