# Supplementary material for: Breeding system and geospatial variation shape the population genetics of Triodanis perfoliata
Source: Ecol Evol. 2022 Oct 8;12(10):e9382. doi: 10.1002/ece3.9382 (PMC9547245; doi:10.1002/ece3.9382)
Supplement: Supplementary file 1 — Table S1 [file ECE3-12-e9382-s003.docx]

Supplemental Table 1. Pairwise F_ST_ values between 18 study populations. Cells are shaded so that darker shades indicate greater values of F_ST_ (greater genetic differentiation).

|  | **NJ** | **WA2** | **NC1** | **KY** | **PA** | **TX** | **WA1** | **VA** | **IL** | **KS2** | **NC2** | **MO** | **KS1** | **CA** | **NY** | **CO** | **OH** | **SC** |
| --- | --- | --- | --- | --- | --- | --- | --- | --- | --- | --- | --- | --- | --- | --- | --- | --- | --- | --- |
| **NJ** | . |  |  |  |  |  |  |  |  |  |  |  |  |  |  |  |  |  |
| **WA2** | 0.623 | . |  |  |  |  |  |  |  |  |  |  |  |  |  |  |  |  |
| **NC1** | 0.448 | 0.676 | . |  |  |  |  |  |  |  |  |  |  |  |  |  |  |  |
| **KY** | 0.385 | 0.484 | 0.45 | . |  |  |  |  |  |  |  |  |  |  |  |  |  |  |
| **PA** | 0.624 | 0.802 | 0.669 | 0.498 | . |  |  |  |  |  |  |  |  |  |  |  |  |  |
| **TX** | 0.51 | 0.582 | 0.542 | 0.395 | 0.638 | . |  |  |  |  |  |  |  |  |  |  |  |  |
| **WA1** | 0.659 | 0.44 | 0.699 | 0.536 | 0.804 | 0.633 | . |  |  |  |  |  |  |  |  |  |  |  |
| **VA** | 0.396 | 0.612 | 0.439 | 0.383 | 0.577 | 0.487 | 0.643 | . |  |  |  |  |  |  |  |  |  |  |
| **IL** | 0.445 | 0.529 | 0.507 | 0.316 | 0.548 | 0.443 | 0.581 | 0.426 | . |  |  |  |  |  |  |  |  |  |
| **KS2** | 0.433 | 0.305 | 0.479 | 0.317 | 0.55 | 0.383 | 0.383 | 0.422 | 0.351 | . |  |  |  |  |  |  |  |  |
| **NC2** | 0.261 | 0.476 | 0.268 | 0.284 | 0.506 | 0.382 | 0.526 | 0.278 | 0.344 | 0.32 | . |  |  |  |  |  |  |  |
| **MO** | 0.609 | 0.78 | 0.656 | 0.459 | 0.762 | 0.597 | 0.789 | 0.569 | 0.514 | 0.49 | 0.462 | . |  |  |  |  |  |  |
| **KS1** | 0.485 | 0.383 | 0.533 | 0.365 | 0.612 | 0.437 | 0.458 | 0.469 | 0.406 | 0.009 | 0.364 | 0.559 | . |  |  |  |  |  |
| **CA** | 0.574 | 0.618 | 0.627 | 0.397 | 0.784 | 0.508 | 0.685 | 0.553 | 0.455 | 0.184 | 0.387 | 0.745 | 0.287 | . |  |  |  |  |
| **NY** | 0.55 | 0.793 | 0.612 | 0.538 | 0.773 | 0.641 | 0.795 | 0.575 | 0.581 | 0.544 | 0.414 | 0.776 | 0.607 | 0.773 | . |  |  |  |
| **CO** | 0.526 | 0.489 | 0.581 | 0.38 | 0.703 | 0.471 | 0.565 | 0.507 | 0.43 | 0.157 | 0.369 | 0.656 | 0.228 | 0.341 | 0.697 | . |  |  |
| **OH** | 0.577 | 0.761 | 0.632 | 0.39 | 0.747 | 0.554 | 0.781 | 0.549 | 0.488 | 0.451 | 0.407 | 0.729 | 0.527 | 0.714 | 0.748 | 0.607 | . |  |
| **SC** | 0.43 | 0.745 | 0.505 | 0.363 | 0.759 | 0.51 | 0.773 | 0.459 | 0.454 | 0.414 | 0.208 | 0.738 | 0.49 | 0.628 | 0.728 | 0.528 | 0.698 | . |
