# Supplementary material for: Breeding system and geospatial variation shape the population genetics of Triodanis perfoliata
Source: Ecol Evol. 2022 Oct 8;12(10):e9382. doi: 10.1002/ece3.9382 (PMC9547245; doi:10.1002/ece3.9382)
Supplement: Supplementary file 2 — Table S2 [file ECE3-12-e9382-s006.docx]

Supplemental Table 2. Pairwise F_ST_ values between K=17 genetic clusters. Note that no individual in our study was assigned the 17th cluster as a primary cluster group. Therefore, this cluster group is not incorporated here. Cells are shaded so that darker shades indicate greater values of F_ST_ (greater genetic differentiation).

|  | **CL1** | **CL2** | **CL3** | **CL4** | **CL5** | **CL6** | **CL7** | **CL8** | **CL9** | **CL10** | **CL11** | **CL12** | **CL13** | **CL14** | **CL15** | **CL16** |
| --- | --- | --- | --- | --- | --- | --- | --- | --- | --- | --- | --- | --- | --- | --- | --- | --- |
| **CL1** | . |  |  |  |  |  |  |  |  |  |  |  |  |  |  |  |
| **CL2** | 0.451 | . |  |  |  |  |  |  |  |  |  |  |  |  |  |  |
| **CL3** | 0.707 | 0.582 | . |  |  |  |  |  |  |  |  |  |  |  |  |  |
| **CL4** | 0.433 | 0.426 | 0.538 | . |  |  |  |  |  |  |  |  |  |  |  |  |
| **CL5** | 0.56 | 0.409 | 0.669 | 0.471 | . |  |  |  |  |  |  |  |  |  |  |  |
| **CL6** | 0.298 | 0.198 | 0.441 | 0.3 | 0.24 | . |  |  |  |  |  |  |  |  |  |  |
| **CL7** | 0.685 | 0.54 | 0.742 | 0.446 | 0.63 | 0.363 | . |  |  |  |  |  |  |  |  |  |
| **CL8** | 0.433 | 0.38 | 0.566 | 0.312 | 0.488 | 0.228 | 0.416 | . |  |  |  |  |  |  |  |  |
| **CL9** | 0.714 | 0.55 | 0.758 | 0.452 | 0.63 | 0.378 | 0.691 | 0.41 | . |  |  |  |  |  |  |  |
| **CL10** | 0.536 | 0.484 | 0.618 | 0.385 | 0.559 | 0.345 | 0.542 | 0.349 | 0.557 | . |  |  |  |  |  |  |
| **CL11** | 0.532 | 0.491 | 0.616 | 0.166 | 0.548 | 0.365 | 0.545 | 0.404 | 0.548 | 0.471 | . |  |  |  |  |  |
| **CL12** | 0.719 | 0.565 | 0.762 | 0.482 | 0.656 | 0.414 | 0.73 | 0.515 | 0.74 | 0.584 | 0.573 | . |  |  |  |  |
| **CL13** | 0.705 | 0.54 | 0.747 | 0.443 | 0.632 | 0.366 | 0.699 | 0.447 | 0.704 | 0.56 | 0.549 | 0.729 | . |  |  |  |
| **CL14** | 0.706 | 0.611 | 0.757 | 0.33 | 0.671 | 0.474 | 0.71 | 0.584 | 0.712 | 0.608 | 0.444 | 0.733 | 0.718 | . |  |  |
| **CL15** | 0.734 | 0.533 | 0.773 | 0.529 | 0.612 | 0.345 | 0.764 | 0.612 | 0.766 | 0.645 | 0.61 | 0.776 | 0.748 | 0.748 | . |  |
| **CL16** | 0.55 | 0.486 | 0.638 | 0.383 | 0.542 | 0.35 | 0.56 | 0.397 | 0.565 | 0.491 | 0.46 | 0.597 | 0.554 | 0.607 | 0.641 | . |
