# Supplementary material for: Breeding system and geospatial variation shape the population genetics of Triodanis perfoliata
Source: Ecol Evol. 2022 Oct 8;12(10):e9382. doi: 10.1002/ece3.9382 (PMC9547245; doi:10.1002/ece3.9382)
Supplement: Supplementary file 3 — Table S3 [file ECE3-12-e9382-s001.docx]

Supplemntal Table 3. Breeding system data for nine populations of *Triodanis perfoliata*, following the methods of Ansaldi et al, 2018a. Breeding systems were only assessed for populations within our larger genetic dataset for which we had access to N>20 fully mature vouchered individuals from a population. On each individual we assessed the total production of CH and CL flowers, and the proportion of flowers that were CH (pCH). Here we report the population averages of these metrics, as well as the sample size (n) of individuals measured. Data for NC2 are derived from Ansaldi et al. 2018a.

| **Pop** | **Total CH** | **Total CL** | **Total Flowers** | **pCH** | **n** |
| --- | --- | --- | --- | --- | --- |
| IL | 21.26 | 18.48 | 39.74 | 0.53 | 27 |
| KS1 | 22.44 | 7.06 | 29.53 | 0.76 | 32 |
| KS2 | 18.85 | 6.52 | 25.41 | 0.74 | 27 |
| KY | 5.28 | 4.7 | 10.16 | 0.52 | 50 |
| MO | 19.82 | 53.45 | 73.27 | 0.27 | 22 |
| NC2 | 8.37 | 6.81 | 15.19 | 0.58 | 70 |
| OH | 13.45 | 21.55 | 35 | 0.4 | 20 |
| PA | 2.29 | 12.46 | 14.75 | 0.16 | 28 |
| TX | 3.85 | 7.78 | 11.63 | 0.33 | 27 |
