# Supplementary material for: Breeding system and geospatial variation shape the population genetics of Triodanis perfoliata
Source: Ecol Evol. 2022 Oct 8;12(10):e9382. doi: 10.1002/ece3.9382 (PMC9547245; doi:10.1002/ece3.9382)
Supplement: Supplementary file 4 — Table S4 [file ECE3-12-e9382-s005.docx]

Supplemental Table 4. Results from a linear model describing the relationship between pCH and mean population pairwise Fst. pCH significantly explains variation in mean pairwise Fst (p=0.0015, multiple R^2^ = 0.78, F = 25.19). Linear models were run in base R (R Core team, 2021) using the formula *lm(dat$pairwise_Fst ~ dat$pCH).*

|  | **Estimate** | **Std. Error** | **t value** | **Pr(>\|t\|)** |
| --- | --- | --- | --- | --- |
| (Intercept) | 0.64949 | 0.04047 | 16.048 | 8.86E-07 |
| pCH | -0.39497 | 0.0787 | -5.019 | 0.00153 |
| Residual standard error | 0.04558 |  |  |  |
