# Supplementary figures and images for: Breeding system and geospatial variation shape the population genetics of Triodanis perfoliata
Source: Ecol Evol. 2022 Oct 8;12(10):e9382. doi: 10.1002/ece3.9382 (PMC9547245; doi:10.1002/ece3.9382)

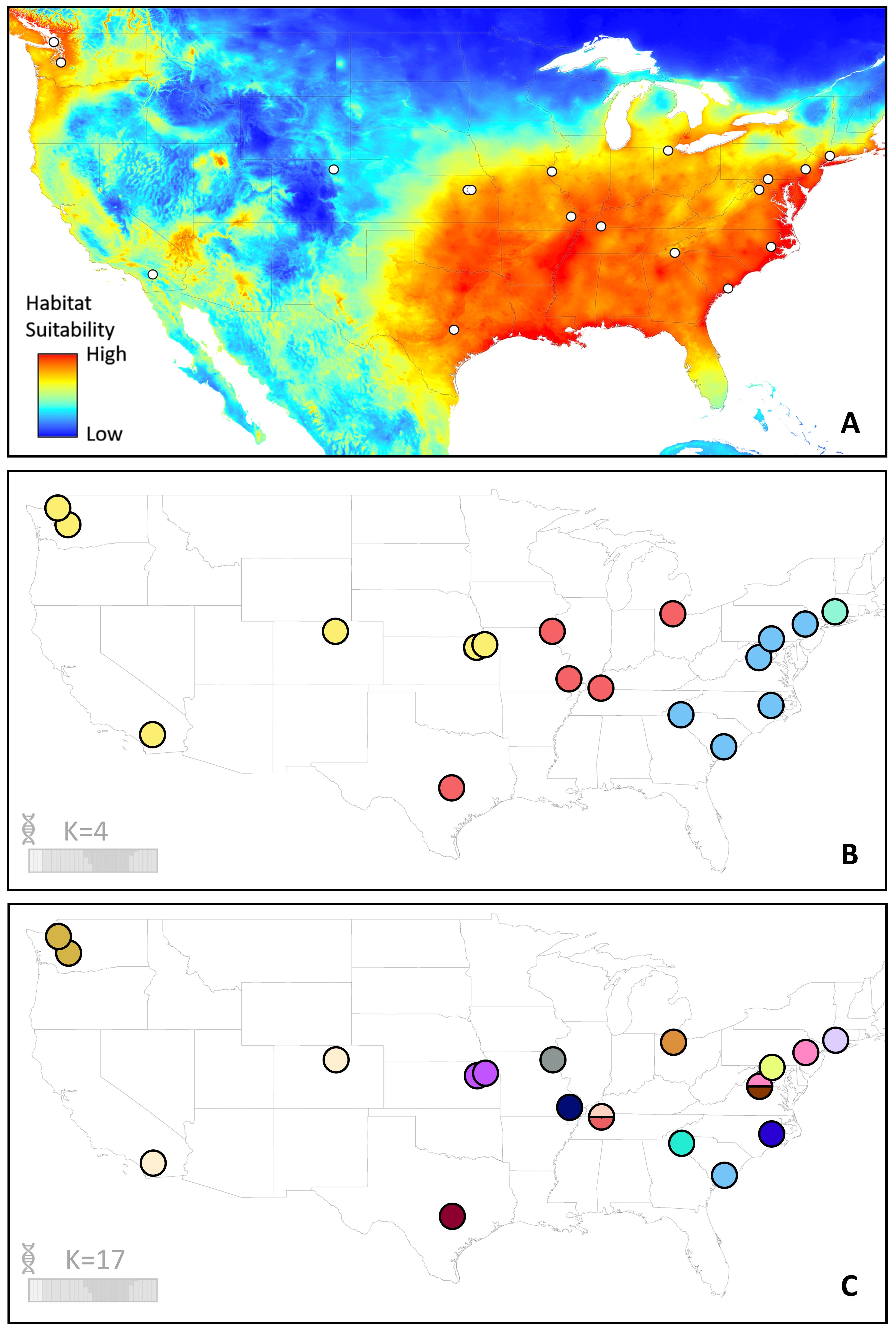

Supplement: Supplementary file 5 — Figure S1 [file ECE3-12-e9382-s002.jpg]
